# A Systematic Review and Network Meta-Analysis on the Impact of Various Aligner Materials and Attachments on Orthodontic Tooth Movement

**DOI:** 10.3390/jfb14040209

**Published:** 2023-04-10

**Authors:** Mohammad Khursheed Alam, Bushra Kanwal, Abedalrahman Shqaidef, Haytham Jamil Alswairki, Ahmed Ali Alfawzan, Abdulilah Ibrahim Alabdullatif, Abdulaziz Naser Aalmunif, Sattam Hamad Aljrewey, Thamer Abdullah Alothman, Deepti Shrivastava, Kumar Chandan Srivastava

**Affiliations:** 1 Orthodontic Division, Preventive Dentistry Department, College of Dentistry, Jouf University, Sakaka 72345, Saudi Arabia; 2 Department of Dental Research Cell, Saveetha Dental College and Hospitals, Saveetha Institute of Medical and Technical Sciences, Chennai 602105, India; 3 Department of Public Health, Faculty of Allied Health Sciences, Daffodil lnternational University, Dhaka 1216, Bangladesh; 4 Practicing in Dental Clinic, Al Baha 65511, Saudi Arabia; 5 Department of Clinical Sciences, Center of Medical and Bio-Allied Health Sciences Research, College of Dentistry, Ajman University, Ajman 346, United Arab Emirates; 6 School of Dental Sciences, Universiti Sains Malaysia, Kota Bharu 16150, Malaysia; 7 Department of Preventive Dentistry, College of Dentistry in Ar Rass, Qassim University, Ar Rass 52571, Saudi Arabia; 8 Ministry of Health, Riyadh 12613, Saudi Arabia; 9 Periodontics Division, Preventive Dentistry Department, College of Dentistry, Jouf University, Sakaka 72345, Saudi Arabia; 10 Oral Medicine & Maxillofacial Radiology Division, Department of Oral & Maxillofacial Surgery & Diagnostic Sciences, College of Dentistry, Jouf University, Sakaka 72345, Saudi Arabia

**Keywords:** 3D printing, aligners, artificial intelligence, future trends, orthodontics, teleorthodontics

## Abstract

The majority of patients strongly favor the use of aligners in the present time, especially with the advancement in esthetic dentistry. Today’s market is flooded with aligner companies, many of which share the same therapeutic ethos. We therefore carried out a systematic review and network meta-analysis to evaluate research that had looked at various aligner materials and attachments and their effect on orthodontic tooth movement in relevant studies. A total of 634 papers were discovered after a thorough search of online journals using keywords such as “Aligners”, “Orthodontics”, “Orthodontic attachments”, “Orthodontic tooth movement”, and “Polyethylene” across databases such as PubMed, Web of Science, and Cochrane. The authors individually and in parallel carried out the database investigation, removal of duplicate studies, data extraction, and bias risk. The statistical analysis demonstrated that the type of aligner material had a significant impact on orthodontic tooth movement. The low level of heterogeneity and significant overall effect further support this finding. However, there was little effect of attachment size or shape on tooth mobility. The examined materials were primarily concerned with influencing the physical/physicochemical characteristics of the appliances and not tooth movement directly. Invisalign (Inv) had a higher mean value than the other types of materials that were analyzed, which suggested a potentially greater impact on orthodontic tooth movement. However, its variance value indicated that there was also greater uncertainty associated with the estimate compared to some of the other plastics. These findings could have important implications for orthodontic treatment planning and aligner material selection. Registration: This review protocol was registered on the International Prospective Register of Systematic Reviews (PROSPERO; registration number: CRD42022381466).

## 1. Introduction

The science of orthodontics is concerned with the diagnosis, prevention, and treatment of dental issues such as jaw malalignment, teeth malalignment, and issues with the process of mastication. The goal of the dentistry specialty known as dentofacial orthopedics, is to correct abnormalities in facial growth [1,2]. Currently, misaligned jaws and teeth are a major problem. The American Association of Orthodontics (AAO) estimates that 50% of people have malocclusions that are severe enough to need orthodontic treatment. According to the same AAO statement [3,4], this percentage falls to less than 10% when implanting orthodontics that are medically required. This important orthodontic problem is being addressed by many researchers who are creating new materials and techniques [5]. During the course of therapy, which could last from a few months to a few years, braces and other appliances will be utilized to gradually realign the teeth and jaws. In cases of severe malocclusion, jaw surgery may be necessary [6,7].

Except for patients who experience adverse reactions, the majority of therapy participants highly favor the aligner [8]. There are many aligner businesses on the market today, many of which have the same therapeutic philosophy. Today, thermoplastic materials, including polyethylene terephthalate glycol-modified (PTG), polypropylene (PP), polycarbonate (PC), thermoplastic polyurethanes (TP), ethylene vinyl acetate, etc., are employed by aligner producers [9]. Materials for correcting tooth placements should be transparent, have a low hardness, good flexibility, and high durability. They also need to be biocompatible and effective [10]. Comfort and aesthetics are the most crucial factors.

Material construction has a significant impact on aligner performance. A total of 50% of the original stress value can be released in the first few hours of wearing an aligner. After 24 h, the aligner’s orthodontic loads and changes in stress have an impact on the planned tooth movement [11]. Although the composition of the aligner materials varied, the thickness of the plates ranged from 0.5 mm to 1.5 mm [12]. The biomechanical characteristics linked to tooth movement can vary depending on the material’s thickness. Thicker aligners deliver greater forces than thinner ones among the various aligner materials [13,14]. Using an aligner treatment to address tooth rotation and torque is challenging [15]. The application of force systems to teeth has been designed into attachments. The various attachment forms are intended to improve retention and enable challenging orthodontic tooth movement. The complexity of optimized attachment forms is rising, and they are demonstrating a therapeutic advantage in managing tooth motions [16]. Beveled attachments have been found to greatly boost retention, whereas ellipsoid attachments have been found to have no discernible impact on retention [17]. Market-available thermoplastic materials have a wide range of mechanical properties. Compared to other materials, PTG materials demonstrated a greater pace of stress relaxation (62% in 24 h) in a 0.75-mm-thick single-layer material stress relaxation investigation. The original stress values for the TPU material were incredibly low.

Therefore, this systematic review and network meta-analysis assessed studies that had examined different aligner materials and attachments and their impact on the orthodontic tooth movement of patients that were undergoing any form of orthodontic treatment and determined whether one type of material was functionally better than the other.

## 2. Materials and Methods

### 2.1. Protocol Employed

**Figure 1:**
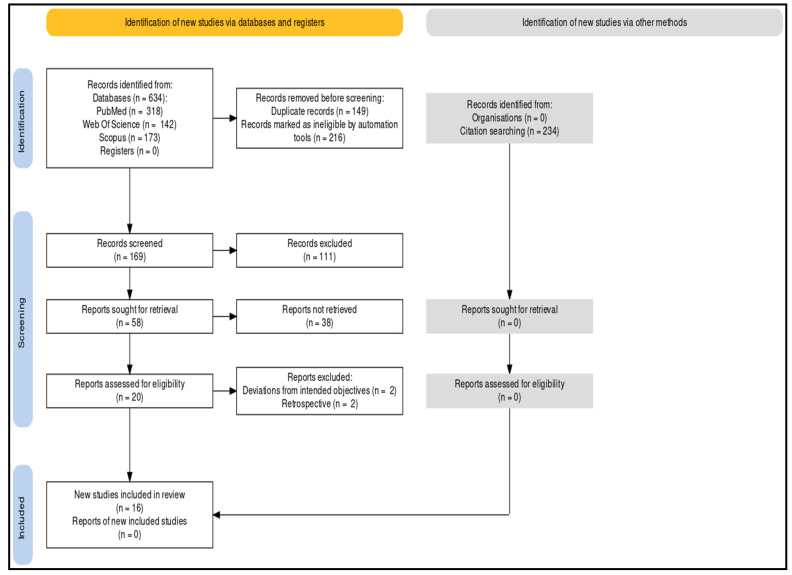
PRISMA protocol of the analysis.

This systematic review was performed as per the Preferred Reporting Items for Systematic Review and Meta-analysis (PRISMA) (Figure 1) strategy and rules from the Cochrane group and the book *Orderly Reviews in Health Care: Meta Examination* [18]. 

### 2.2. Review Hypotheses

Ours was a systematic review and network meta-analysis of research that looked at various aligner materials and attachments and their effects on the orthodontic tooth movement of either actual patients or inanimate objects.

### 2.3. Inclusion Criteria

For full-text screening, articles that included information pertinent to our review goals were chosen. We took into consideration for inclusion in our review studies those that reported clinical trials, in vitro investigations, randomized/non-randomized studies, systematic literature reviews with sizable sample sizes, and comprehensive case reports. We also kept an eye on papers with better methodological quality.

### 2.4. Exclusion Criteria

Studies that were primarily seminar-based articles, academic papers, editorials, and data with gaps were excluded from our thorough study.

We considered all publications that had been written about our topic; we did not restrict our search by the studies’ publication dates. This is due to the paucity of literature on the subject in relation to the goal of our investigation.

The analysis was not performed on studies that employed placebos. Any reviews of the literature or case studies that were written in a language other than English were also ignored.

### 2.5. Search Strategy

The Cochrane, PubMed, and Web of Science databases were all searched using pertinent keywords, reference searches, and citation searches. Among the search phrases used to access the database were “Aligners”, “Orthodontics”, “Orthodontic attachments”, “Orthodontic tooth movement”, and “Polyethylene”.

The PICO strategy that we utilized for the current review was as follows: Population: Patients undergoing orthodontic treatment with clear aligners; Intervention: Various aligner materials and attachments; Comparison: Conventional clear aligners or different types of aligner materials and attachments and Outcome: Orthodontic tooth movement, including rate, amount, and direction.

So, the complete PICO strategy using Boolean operators was something such as this: (“orthodontic treatment” AND “clear aligners”) AND (“aligner materials” OR “attachments”) AND (“conventional clear aligners” OR “different types of aligner materials” OR “different types of attachments”) AND (“orthodontic tooth movement” AND (“rate” OR “amount” OR “direction”)).

### 2.6. Data Selection and Coding

Using the relevant keywords in multiple databases and internet search tools, two separate reviewers found the pertinent papers. A third reviewer was consulted if there was disagreement after comparing the selected articles.

The same two reviewers independently extracted the data pertinent to the goals of our investigation after selecting the papers. After comparing the data, the third reviewer discussed any discrepancies.

### 2.7. Statistical Analysis

A web tool called CINeMA (Confidence in Network Meta-Analysis) makes it easier to assess one’s level of confidence in the results of network meta-analysis. It is based on a methodological paradigm that takes six areas into account: incoherence, indirectness, imprecision, heterogeneity, and within-study bias. The contribution matrix, which demonstrates how much information each study adds to the outcomes of network meta-analysis, is a key component of the CINeMA technique [19]. Apart from these, forest plots of odds ratios and risk ratios representing the effects of different aligner materials on orthodontic tooth movement were obtained using the RevMan 5 software.

### 2.8. Risk of Bias Assessment in Individual Studies

The AMSTAR-2 approach was used to evaluate the studies we chose for Table 1 for bias [20]. The domains listed in the Cochrane risk of bias instruments for systematic reviews are identified by the AMSTAR 2 risk of bias items. These show that a choice was made in each case following input from more than 30 methodological experts.

## 3. Results

### 3.1. Study Selection

A total of 634 documents in all were found following a thorough search of the online journals, whereas another 234 were identified from other methods such as citations of included studies and various bibliographic sources. Of these, 169 of the papers were first chosen. Then, 111 similar/duplicate publications were removed, leaving 58 distinct papers that were initially available. After reviewing the submissions’ abstracts and titles, 42 more articles were disqualified. Ultimately, 16 documents were selected that satisfied the necessary inclusion and exclusion criteria, primarily in vitro experiments, literature reviews, and comparative analyses.

### 3.2. Study Characteristics

**Figure 2:**
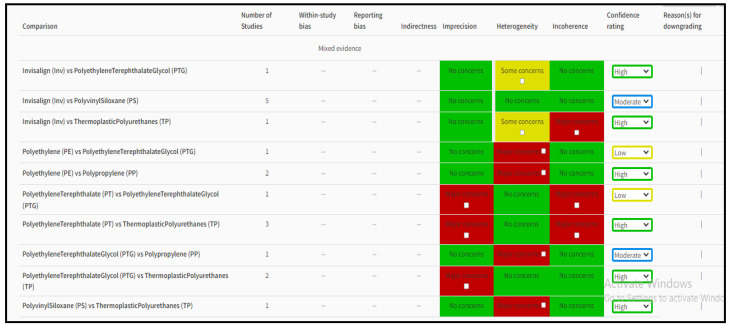
Assessment of risk of bias of various treatment modalities using the CiNeMA tool.

**Figure 3:**
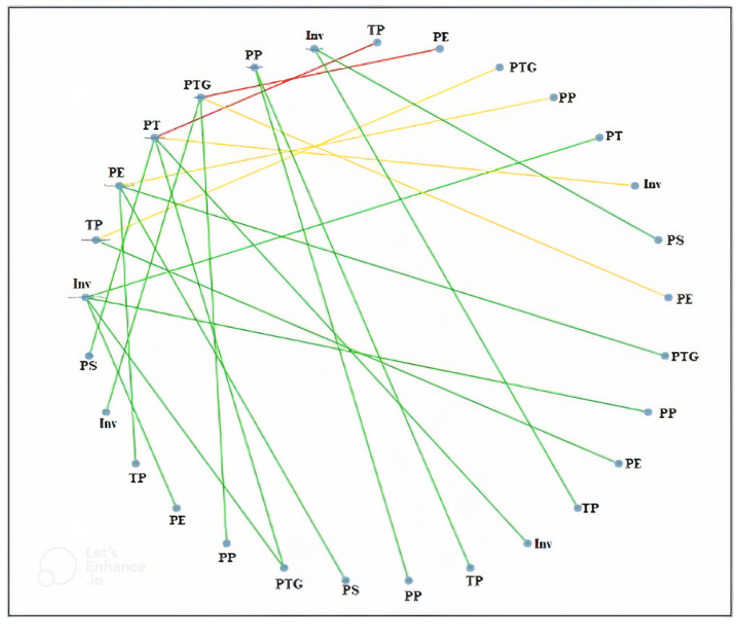
Correlation between all the treatment modalities and the results of the network meta-analysis of various treatment modalities using the CiNeMA tool [where Invisalign (Inv), Polyethylene (PE), PolyethyleneTerephthalate (PT), PolyethyleneTerephthalateGlycol (PTG), Polypropylene (PP), PolyvinylSiloxane (PS), and ThermoplasticPolyurethanes (TP)].

Table 2 lists the study’s design, methods, description, and results. Figure 2 and Figure 3 present the network meta-findings’ analysis. Out of the 16 studies that we selected for the systematic review and subsequent meta-analysis, 1 was a comparative clinical trial [24], 2 were prospective studies [22,29], and the rest were in vitro studies that investigated different aligner materials [10,11,13,14,23,25,26,27,28,30,31,32]. In the clinical trial by Ercoli et al. [24], the study compared the Nuvola^®^ and Fantasy^®^ systems, looked at their material characteristics, and identified when to employ aligners. The Nuvola^®^ aligner and the Fantasmino^®^ system were used to treat two different patient groups. In the two prospective studies by D’Anto et al. and Kravitz et al., respectively [24,29], the methodology employed was somewhat similar. In both studies, to compare the virtually intended and the clinical tooth position, prescription, achieved movement, and performance measures were computed.

The values represented in Table 3 provide the posterior distributions of various parameters for different plastics used in the study of the impact of various aligner materials and attachments on orthodontic tooth movement. The mean values indicate the central tendency of the posterior distribution for each plastic material. The mean values for Polyethylene (PE) and PolyethyleneTerephthalate (PT) are 216.941 and 85.353, respectively. These values provide an estimate of the average impact of these materials on orthodontic tooth movement. The variance values for each plastic indicate the spread of the posterior distribution around the mean. Higher variance values suggest that the data points are more spread out from the mean, indicating greater uncertainty in the estimates. The variance values for Polyethylene (PE), PolyethyleneTerephthalate (PT), PolyethyleneTerephthalateGlycol (PTG), Polypropylene (PP), and PolyvinylSiloxane (PS) are 280,205.627, 560,411.255, 272,199.752, 272,199.752, and 176,425.765, respectively. These variance values suggest that the estimates for PT and PTG have greater uncertainty compared to the other materials.

The lower and upper bounds for each material indicate the range of possible values that the true parameter value could fall within, based on the posterior distribution. For example, the lower bound for PolyethyleneTerephthalate (PT) is −1392.676 and the upper bound is 1563.382. These bounds provide an estimate of the range of values for the impact of PT on orthodontic tooth movement. These values provide estimates of the impact of different plastics on orthodontic tooth movement and the uncertainty associated with these estimates. However, it is important to note that these estimates are based on a specific study design and may not be generalizable to other settings. Further research is needed to confirm these findings and to explore the impact of other factors on orthodontic tooth movement.

Compared to the other plastics, Invisalign (Inv) has a higher mean value of 642.467, which suggests a potentially greater impact on orthodontic tooth movement. However, the variance value of 158,783.189 indicates that there is also greater uncertainty associated with this estimate compared to some of the other plastics. The lower bound of −144.274 and the upper bound of 1429.208 suggest a wide range of possible values for the impact of Invisalign on orthodontic tooth movement, with a 95% confidence level. This range of values reflects the uncertainty associated with the estimate and highlights the need for further research to confirm the impact of Invisalign on orthodontic tooth movement.

Overall, the values for Invisalign are comparable to the other plastics in terms of the range of estimates and the uncertainty associated with the estimates. Further research is needed to better understand the impact of different aligner materials and attachments on orthodontic tooth movement.

Figure 3 represents the results of the network meta-analysis that we obtained after entering data on the various aligner materials that were observed in the studies we selected for the review. It reveals the interconnectedness of all the types of aligner materials, indicating that the materials performed on a similar basis with not a very noticeable difference separating their effects.

**Figure 4:**
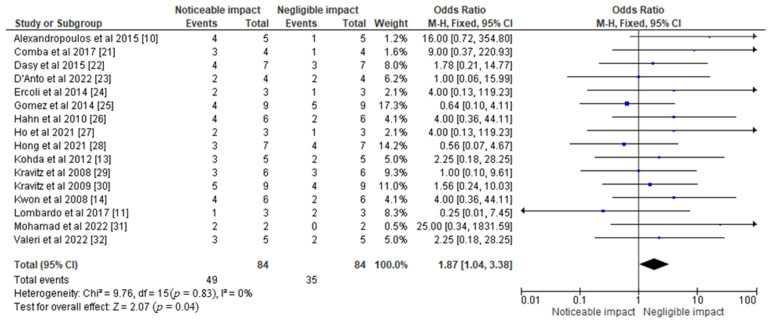
Forest plot representing the odds ratio of different aligner materials observed in the selected studies and their respective impact on orthodontic tooth movement [10,11,13,14,21,22,23,24,25,26,27,28,29,30,31,32].

The forest plot in Figure 4 presents the results of a statistical analysis of the impact of different aligner materials on orthodontic tooth movement. The odds ratio value of 1.87 [1.04, 3.38] was obtained from the selected studies, indicating a significant association between aligner materials and orthodontic tooth movement. The analysis showed that the odds of experiencing orthodontic tooth movement were 1.87 times higher in patients treated with certain aligner materials compared to those treated with other materials. The analysis also indicated a low level of heterogeneity (I^2^ = 0%), suggesting that the selected studies were consistent in their findings. The chi-square value of 9.76 with 15 degrees of freedom (*p* = 0.83) also supported this conclusion. The test for overall effect yielded a Z value of 2.07 (*p* = 0.04), indicating that the results were statistically significant. This suggests that aligner materials have a significant impact on orthodontic tooth movement.

**Figure 5:**
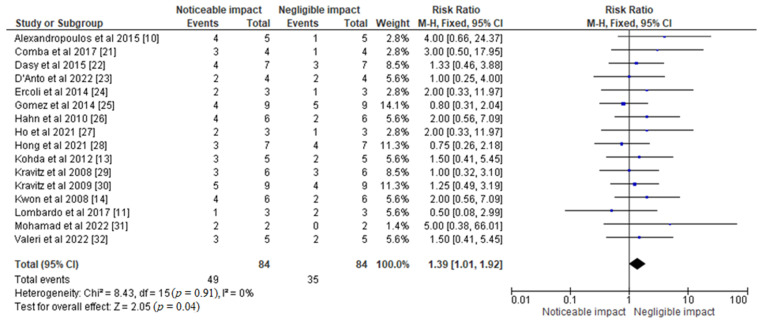
Forest plot representing the risk ratio of different aligner materials observed in the selected studies and their respective impact on orthodontic tooth movement [10,11,13,14,21,22,23,24,25,26,27,28,29,30,31,32].

The forest plot, as seen in Figure 5, shows the risk ratio of different aligner materials on orthodontic tooth movement based on the selected studies. The pooled estimate of the effect size was 1.39 [1.01, 1.92], with a fixed effects model. The test for overall effect was statistically significant, with a Z-value of 2.05 and a *p*-value of 0.04. The forest plot indicates that the effect sizes of the individual studies ranged from 0.89 to 2.08, with a 95% confidence interval. The heterogeneity of the studies was low, with a Chi^2^ value of 8.43, a *p*-value of 0.91, and an I^2^ value of 0%. These findings suggest that different aligner materials have a significant impact on tooth movement. However, the effect size of each study varied, which may be due to differences in study design, sample size, and other factors.

Overall, comparing Invisalign to the other types of materials analyzed, it had a higher mean value, which indicated a potentially greater impact on orthodontic tooth movement. However, the values for Invisalign were comparable to those for the other plastics in terms of the range of estimates and the degree of ambiguity surrounding the estimates. More research is needed to fully comprehend how different aligner materials and components affect the movement of orthodontically aligned teeth. Moreover, compared to some of the other plastics, Invisalign’s variance number showed that the estimate also had a higher level of uncertainty. These results indicate that the use of different aligner materials should be carefully considered in orthodontic treatment planning.

### 3.3. Risk of Bias Assessment within the Study

The studies we selected for Table 1 were assessed for bias using the AMSTAR-2 method [20]. The AMSTAR 2 risk of bias items identifies the domains specified in the Cochrane risk of bias instruments for systematic reviews. These demonstrate that decisions were made in each case after considering the opinions of more than 30 methodological experts. Apart from this, the overall quality of the available evidence using the GRADE Pro software [GRADEpro GDT: GRADEpro Guideline Development Tool. McMaster University and Evidence Prime, 2022] was judged to be moderate for the in vitro studies and the prospective clinical studies [10,11,13,14,22,23,25,26,27,28,29,30,31,32] that analyzed the effects of aligner materials and attachments on orthodontic activity, and high for the one clinical trial [24] that was included in the review.

The CINeMA-based results of network meta-analysis of various treatment modalities observed in selected studies are shown in Table 3. Apart from these, forest plots of odds ratios and risk ratios representing the effects of different aligner materials on orthodontic tooth movement were obtained using the RevMan 5 software, as represented in Figure 4 and Figure 5, respectively.

## 4. Discussion

The significance of this study is that it provides an in-depth analysis of the impact of different aligner materials and attachments on orthodontic tooth movement, including the rate, amount, and direction of movement. By using a systematic review and meta-analysis approach, the study has synthesized the findings of multiple studies to draw robust conclusions about the effects of different aligner materials and attachments on orthodontic tooth movement. We also used advanced statistical methods, such as network meta-analysis and posterior distributions, to provide a more nuanced understanding of the impact of different aligner materials and attachments on orthodontic tooth movement, and as far as the literature is concerned, we did not encounter any network meta-analysis that investigated the same objectives that we did. Overall, the findings of this study have implications for orthodontic practice, as they suggest that the choice of aligner material and attachment can significantly impact orthodontic tooth movement. The study highlights the need for clinicians to carefully consider the type of aligner material and attachment they use for each patient, considering factors such as the severity of the malocclusion and the desired outcome. Additionally, the study provides a foundation for future research in this area by identifying gaps in current knowledge and suggesting avenues for further investigation. The values for Invisalign were similar to the other plastics in terms of the range of estimates and uncertainty associated with the estimates, according to the results from the network meta-analysis. To better understand the effect of various aligner materials and attachments on orthodontic tooth movement, more studies are required.

By altering the curvature of the aligner, the teeth can be moved. Inconsistencies in the aligners may produce the tipping force required to shift the teeth. This tipping force pushes against the clinical crown, but not against the tooth center resistance. As a result, the movement of the aligner tooth always causes the crown tip to move. Two variables must be considered in order to improve aligner retention. The first is the application and shape of the attachment, and the second is the material choice for the aligner [17]. In order to increase the ability of orthodontic aligners to move the tooth, forces or movements are generated using a precise geometric attachment [22]. Beveled attachments demand more force to remove an aligner from a cast than ellipsoid attachments or models with no attachment, whether they are beveled or not. Retention is influenced by the nature of the material and is not always correlated with material thickness [25].

Ho et al. [26] evaluated the impact of the attachment geometry on tooth movement in their investigation using the same thickness of aligner material. In this investigation, there was more crown tipping as a result of the aligner bar-shaped attachment. To ascertain the impact of attachment on tooth movement, the PTG and TP group aligners with and without attachment were compared. There were no differences in tooth movement outcomes between the PTG and TP groups based on attachment. This might have happened as a result of the low Young’s moduli of the aligners employed in the PTG or TP groups being unable to tolerate the stress caused by tooth tipping, which in turn caused tooth tipping. In the current investigation, the ellipsoid and bar shapes were used as shape attachments. The ellipsoid attachment was positioned on the distal occlusal site and on the mesial gingival site of the canine crown and had two contour thicknesses, one thin and one thick. It was intended to rub up against the crown’s distal tip. To counteract the distal movement force causing root mesial tipping, the bar attachment was created as a long, rectangular shape that was half the clinical length. Two points of contact and a large area of contact to operate against the aligner tipping force distinguished the ellipsoid from the bar shape [21]. With both ellipsoid- and bar-shaped attachments, the PTG or TP group aligners corrected the canine’s crown distal tipping and root mesial torque. The PTG group demonstrated that, compared to the ellipsoid attachment group, the bar type attachment induced a higher canine long axis angle. In other words, there was more canine distal tilting in the group of PTG bar attachments. On the canine of the PTG group, using the thin or thick ellipsoid attachment resulted in tooth distal tipping but no statistically significant difference in the canine’s long axis angle. This demonstrated that crown tipping was not prevented by an ellipsoid or bar-shaped attachment [25].

Two variables must be considered in order to improve aligner retention. The first is the application and shape of the attachment, and the second is the material choice for the aligner [23]. In order to increase the ability of orthodontic aligners to move the tooth, forces or movements are generated using a precise geometric attachment. Beveled attachments demand more force to remove an aligner from a cast than ellipsoid attachments or models with no attachment, whether they are beveled or not. Retention is influenced by the nature of the material and is not always correlated with material thickness [26]. The ellipsoid attachment was preferred above the bar attachment in the current study to reduce canine distal tilting. Canine tipping was not prevented by the attachment. The orthodontic power and moment produced by the attachment may not have been sufficient to move his teeth in the way he had intended [29].

## 5. Limitations

Our study did not include any significant percentage of randomized clinical trials, which could be said to be a major drawback, and the fact that the overall number of investigations that we selected as well as their respective sample sizes for assessment and subsequent meta-analysis might be deemed to be less than ideal. However, the fact is that there was a dearth of investigations that could be found assessing the effects of the aligner materials used in routine practice on orthodontic tooth movement, as mentioned in our selected studies. Additionally, a large portion of the literature devoted to these kinds of creative approaches was limited to scoping/literature reviews. So, in order to establish definite therapeutic approaches adjuvant to traditional orthodontic treatment, we believe more studies are needed in this regard.

## 6. Conclusions

In conclusion, the goal of the current study was to identify the influence of orthodontic tooth movement on aligner behavior by choosing trials that stimulated both human participants and in vitro settings. The findings suggested that aligner materials had a significant impact on tooth movement, but not in terms of their attachment size and shape but rather the type of material that was used. Within the types of materials that were assessed, not much of a noticeable difference was observed; however, as per the findings obtained through the network meta-analysis, the values for Invisalign are comparable to the other plastics in terms of the range of estimates and the uncertainty associated with the estimates. Further research is needed to better understand the impact of different aligner materials and attachments on orthodontic tooth movement. Further studies in this regard, where different orthodontic materials and their impact on tooth movement are assessed, may shed more light on this phenomenon.

## Figures and Tables

**Table 1:** AMSTAR-2 16-point checklist of risk of bias assessment in studies selected for the systematic review.

Studies Selected	Question and Inclusion	Protocol	Study Design	Comprehensive Search	Study Selection	Data Extraction	Excluded Studies Justification	Included Study Details	Risk of Bias	Funding Sources	Statistical Methods	Risk of Bias in Meta-Analysis	Risk of Bias in Individual Studies	Explanation of Heterogeneity	Publication Bias	Conflict of Interest
Alexandropoulos et al., 2015 [10]	Yes	Yes	Yes	Yes	Yes	No	No	No	Yes	N/A	Yes	Yes	Yes	Yes	Yes	Yes
Comba et al., 2017 [21]	Yes	Yes	Yes	Yes	Yes	No	No	No	Yes	N/A	Yes	Yes	Yes	Yes	Yes	Yes
D’Anto et al., 2022 [22]	Yes	Yes	Yes	Yes	Yes	No	No	No	Yes	N/A	Yes	N/A	Yes	Yes	Yes	Yes
Dasy et al., 2015 [23]	Yes	Yes	Yes	Yes	Yes	No	No	No	Yes	N/A	Yes	Yes	Yes	Yes	Yes	Yes
Ercoli et al., 2014 [24]	Yes	Yes	Yes	Yes	Yes	No	No	No	Yes	Yes	Yes	Yes	Yes	Yes	Yes	Yes
Gomez et al., 2014 [25]	Yes	Yes	Yes	Yes	Yes	No	No	No	Yes	Yes	Yes	Yes	Yes	Yes	Yes	Yes
Hahn et al., 2010 [26]	Yes	Yes	Yes	Yes	Yes	No	No	No	Yes	N/A	Yes	Yes	Yes	Yes	Yes	Yes
Ho et al., 2021 [27]	Yes	Yes	Yes	Yes	Yes	No	No	No	Yes	N/A	Yes	Yes	Yes	Yes	Yes	Yes
Hong et al., 2021 [28]	Yes	Yes	Yes	Yes	Yes	No	No	No	Yes	N/A	Yes	N/A	Yes	Yes	Yes	Yes
Kohda et al., 2012 [13]	Yes	Yes	Yes	Yes	Yes	No	No	No	Yes	N/A	Yes		Yes	Yes	Yes	Yes
Kravitz et al., 2008 [29]	Yes	Yes	Yes	Yes	Yes	No	No	No	Yes	N/A	Yes	Yes	Yes	Yes	Yes	Yes
Kravitz et al., 2009 [30]	Yes	Yes	Yes	Yes	Yes	No	No	No	Yes	N/A	Yes	Yes	Yes	Yes	Yes	Yes
Kwon et al., 2008 [14]	Yes	Yes	Yes	Yes	Yes	No	No	No	Yes	N/A	Yes	N/A	Yes	Yes	Yes	Yes
Lombardo et al., 2017 [11]	Yes	Yes	Yes	Yes	Yes	No	No	No	Yes	N/A	Yes	Yes	Yes	Yes	Yes	Yes
Mohamad et al., 2022 [31]	Yes	Yes	Yes	Yes	Yes	No	No	No	Yes	Yes	Yes	Yes	Yes	Yes	Yes	Yes
Valeri et al., 2022 [32]	Yes	Yes	Yes	Yes	Yes	No	No	No	Yes	N/A	Yes	Yes	Yes	Yes	Yes	Yes

**Table 2:** Description and outcomes as observed in the studies selected for the systematic review.

Author and Year of Study	Study Design	Study Description/Characteristics	Study Outcome/Inference
Alexandropoulos et al., 2015 [10]	In vitro study	The current study’s objective was to describe the mechanical and chemical characteristics of modern thermoplastic orthodontic materials. Tests were conducted on Clear Aligner (Scheu-Dental), ACE and A+ (Dentsply), and Invisalign, four thermoplastic materials (Align Technology). Each material was used to create eight appliances, and a small sample of each was used to conduct an ATR-FTIR spectroscopy analysis.	Invisalign’s polyurethane base was discovered through ATR-FTIR analysis, while the other materials were based on polyester and polyethylene glycol terephthalate. In comparison to PETG-based products, Invisalign displayed higher hardness and modulus values, a slightly higher brittleness, and lower creep resistance.
Comba et al., 2017 [21]	In vitro study	The force system and displacement patterns produced by plastic aligners during bodily canine movement, both with and without composite attachments and Class II elastics, were described in this work using a finite element (FE) model.	The findings demonstrated that a vertical, rectangular attachment resulted in buccal displacement of the tooth and potential periodontal damage. Movements of intrusion and tipping were produced by configurations with and without an attachment.
D’Anto et al., 2022 [22]	Prospective study	This study compared the virtually intended and actual tooth movement at the end of stage 15, which is frequently the moment of initial refinement, in order to assess the predictability of CAT.	The first molars’ tip correction had the most under-performance (+2.3° ± 3.1°), the second molars’ torque correction had the largest over-performance (+2.3° ± 3.1°), and rotation corrections of all the teeth had the best accuracy.
Dasy et al., 2015 [23]	In vitro study	This study’s primary objective was to assess how well four different types of aligners stayed in place on a dental arch with different attachments. Three casts—one without any attachments to act as a control—were produced for this investigation.	Except when utilized with attachments, CA-soft, CA-medium, and CA-hard did not significantly boost retention. Furthermore, CA-hard and CA-medium required a great deal more energy to remove.
Ercoli et al., 2014 [24]	Comparative clinical trial	This research compared the Nuvola^®^ and Fantasy^®^ systems, looked at their material characteristics, and identified when to employ aligners. The Nuvola^®^ aligner and the Fantasmino^®^ system were used to treat two different patient groups.	Throughout the course of the therapy, the two types of aligners demonstrated variances. Although the Fantasmino^®^ system has highly effective elastic qualities, its size did not promote compliance throughout the day.
Gomez et al., 2014 [25]	In vitro study	The purpose of this study was to describe the initial force system produced during physical movement of upper canines with plastic aligners that had and did not have composite attachments using a three-dimensional finite element (FE) model. A thermoformed plastic aligner, two light-cured composite attachments, and a CAD model of an upper right canine’s alveolar bone and periodontal ligament were created.	Without composite attachments, it was possible to see a compression area in the cervical third of the distal root surface and a tension area in the apical third of the mesial surface in terms of the stress distribution between tension and compression.
Hahn et al., 2010 [26]	In vitro study	The purpose of this study was to quantify the stresses and moments applied by three identically thick thermoplastic appliances to a maxillary central incisor during rotation. The three materials were used to create five identical appliances (Ideal Clear 1.0 mm, Erkodur 1.0 mm, and Biolon 1.0 mm).	The least moment was measured at a rotational speed of 20.17 mm (27.3 Nmm, 60.8), while the largest moment was measured at a deflection speed of 20.51 mm (271.8 Nmm, 62.5). The lowest intrusive force was measured at activation of 20.17 mm (0.0 N), while the highest intrusive force was assessed at rotation of 20.51 mm (25.8 N).
Ho et al., 2021 [27]	In vitro study	This study examined the behavior of a single tooth moving in an orthodontic aligner utilizing various aligner materials and attachment shapes. The first typodont models were created using a 3D printer and bicuspid extracted resin. Three different attachment types—a thick and thin ellipsoid and a bar—were created to fit the canine crown surface. Different aligners were made from three different types of aligner materials.	The BENQ group had a lesser shift in the long axis angle when the three aligners were compared, according to the changes in the canine’s long axis. The canine movement of the BENQ group did not exhibit noticeable movement, but the tipping canine movement of the PTG and TPU groups did.
Hong et al., 2021 [28]	In vitro study	By comparing and contrasting the movement and rotation of teeth between a general attachment and an overhanging attachment, the authors of this study developed an attachment design that effectively induces tooth movement.	The outcomes demonstrated that the overhanging attachment aligner can successfully lessen crown tipping and prevent axial rotation for a central incisor’s targeted distal displacement.
Kohda et al., 2012 [13]	In vitro study	This study’s primary objectives were to quantify the forces applied by thermoplastic appliances constructed of three different materials and examine the impact of mechanical characteristics, material thickness, and activation level on these forces. We chose three thermoplastic materials with two different thicknesses.	Hardcast’s elastic modulus and hardness were much lower than those of Duran and Erkodur, whose characteristics were similar. Appliances made of thicker material (0.75 mm or 0.8 mm) consistently produced a lot more force than those made of thinner material (0.4 mm or 0.5 mm).
Kravitz et al., 2008 [29]	Prospective study	This study’s objective was to assess how attachments and interproximal reduction affected dogs receiving Invisalign rotational therapy. In this prospective clinical investigation, the virtual TREAT models of 31 people who had anterior Invisalign treatment were used to quantify 53 canines (33 maxillary and 20 mandibular).	With Invisalign, the average canine rotation accuracy was 35.8% (SD 26.3). According to statistical analyses, there was no appreciable distinction in accuracy between groups AO, IO, and N.
Kravitz et al., 2009 [30]	In vitro study	A total of 37 patients undergoing anterior Invisalign treatment were included in the study sample. On the virtual Treat models, measurements of 400 anterior teeth (198 maxillary and 203 mandibular) were taken.	With Invisalign, tooth mobility was 41% more accurate on average. Lingual constriction was the movement that was most correct (47.1%), and extrusion (29.6%)—specifically, extrusion of the maxillary (18.3%) and mandibular (24.5%) central incisors, followed by mesiodistal tilting of the mandibular canines (26.9%)—was the action that was least precise.
Kwon et al., 2008 [14]	In vitro study	This study’s goals were to assess the force and energy (resilience) delivery characteristics of thermoplastic overlay orthodontic materials and to identify how these characteristics changed following thermocycling or repeated load cycling. Materials of three sorts and three thicknesses were examined.	Amounts of 0.2 to 0.5 mm of deflection was required for the best force delivery. In the deflection range of the best force delivery, thin material exerted high energy. The force delivery properties after thermocycling were not different from those at the baseline but were different after repeated load cycling in the deflection ranges of optimal force delivery (0.2–0.5 mm).
Lombardo et al., 2017 [11]	In vitro study	This study’s major objective was to look into how 4 thermoplastic polymers used to make orthodontic aligners released stress after being deflected for 24 h straight. Two single-layer and two double-layer aligner materials were chosen.	Throughout the 24-h timeframe, all polymers examined produced a sizable amount of stress. The first 8 h had the greatest relief from stress, establishing a plateau that largely stayed steady.
Mohamad et al., 2022 [31]	In vitro study	This study detailed how the force in clear aligner attachments was topographically visualized. In an in vitro study employing resin models, the authors described a way for obtaining the topographical visualization of a clear aligner’s distributed force on the attachment using Prescale R pressure film and an image processing technology.	The average force for active aligners was between 6.2 and 6.3 N, while the average force for passive aligners was between 4.8 and 4.9 N. Therefore, during this study, a single transparent aligner attachment in a resin model received a net force of 1.3–1.4 N.
Valeri et al., 2022 [32]	In vitro study	The objective of this study was to evaluate the precision of the attachment bonding procedure used in aligner treatments. The authors used two transfer templates made of two different types of materials; the first, known as Leone-biocompatible thermoforming material hard/soft, had a lower Young’s modulus and was designated as soft, while the second, known as Leone-biocompatible thermoforming material, was designated as rigid.	From first with the lowest reproduction error to last with the worst performance, the data processing assigned the following performance ranking: C-Transbond, A-Transbond, C-Evoflow, and A-Evoflow are the first four. Contrary to popular belief, employing a rigid or flexible transfer template had less of an impact than using resin-based composites with various rheologies.

**Table 3:** Statistical analysis using the CiNeMa tool of various treatment modalities observed in selected studies.

Bayesian Estimates of Coefficients ^a,b,c,d^				
Parameter	Posterior		95% Credible Interval	
	Mean	Variance	Lower Bound	Upper Bound
Invisalign (Inv)	642.467	158,783.189	−144.274	1429.208
Polyethylene (PE)	216.941	280,205.627	−828.183	1262.066
PolyethyleneTerephthalate (PT)	85.353	560,411.255	−1392.676	1563.382
PolyethyleneTerephthalateGlycol (PTG)	1304.771	272,199.752	274.685	2334.857
Polypropylene (PP)	97.886	272,199.752	−932.200	1127.972
PolyvinylSiloxane (PS)	1350.259	176,425.765	520.961	2179.557
ThermoplasticPolyurethanes (TP)	1011.200	238,174.783	47.643	1974.757

^a^. Dependent Variable: Young’s modulus as observed in each study. ^b^. Model: Type of aligner material/attachment used in our selected studies. ^c^. Regression Weight Variable: Study ID. ^d^. Assume standard reference priors.

## Data Availability

The authors confirm that the data supporting the findings of this study are available within the article.
